# Development of machine learning algorithms to predict viral load suppression among HIV patients in Conakry (Guinea)

**DOI:** 10.3389/frai.2025.1446876

**Published:** 2025-03-19

**Authors:** Degninou Yehadji, Geraldine Gray, Carlos Arias Vicente, Petros Isaakidis, Abdourahimi Diallo, Saa Andre Kamano, Thierno Saidou Diallo

**Affiliations:** ^1^Médecins Sans Frontières Belgique, Guinea Mission, Conakry, Guinea; ^2^Technological University Dublin, School of Informatics and Cybersecurity, Dublin, Ireland; ^3^Médecins Sans Frontières, Southern Africa Medical Unit, Cape Town, South Africa; ^4^University of Ioannina School of Medicine, Department of Hygiene and Epidemiology, Clinical and Molecular Epidemiology Unit, Ioannina, Greece; ^5^Ministry of Health and Public Hygiene, National HIV and Hepatitis Control Program (PNLSH), Conakry, Guinea

**Keywords:** HIV, antiretroviral therapy, viral load, machine learning, prediction, classification, algorithm

## Abstract

**Background:**

Viral load (VL) suppression is key to ending the global HIV epidemic, and predicting it is critical for healthcare providers and people living with HIV (PLHIV). Traditional research has focused on statistical analysis, but machine learning (ML) is gradually influencing HIV clinical care. While ML has been used in various settings, there’s a lack of research supporting antiretroviral therapy (ART) programs, especially in resource-limited settings like Guinea. This study aims to identify the most predictive variables of VL suppression and develop ML models for PLHIV in Conakry (Guinea).

**Methods:**

Anonymized data from HIV patients in eight Conakry health facilities were pre-processed, including variable recoding, record removal, missing value imputation, grouping small categories, creating dummy variables, and oversampling the smallest target class. Support vector machine (SVM), logistic regression (LR), naïve Bayes (NB), random forest (RF), and four stacked models were developed. Optimal parameters were determined through two cross-validation loops using a grid search approach. Sensitivity, specificity, predictive positive value (PPV), predictive negative value (PNV), *F*-score, and area under the curve (AUC) were computed on unseen data to assess model performance. RF was used to determine the most predictive variables.

**Results:**

RF (94% *F*-score, 82% AUC) and NB (89% *F*-score, 82% AUC) were the most optimal models to detect VL suppression and non-suppression when applied to unseen data. The optimal parameters for RF were 1,000 estimators and no maximum depth (Random state = 40), and it identified Regimen schedule_6-Month, Duration on ART (months), Last ART CD4, Regimen schedule_Regular, and Last Pre-ART CD4 as top predictors for VL suppression.

**Conclusion:**

This study demonstrated the capability to predict VL suppression but has some limitations. The results are dependent on the quality of the data and are specific to the Guinea context and thus, there may be limitations with generalizability. Future studies may be to conduct a similar study in a different context and develop the most optimal model into an application that can be tested in a clinical context.

## Introduction

Human immunodeficiency virus (HIV) has become one of the global health and development challenges since its recognition and report of first cases in the 1980s, and its impacts include social, cultural, psychological, economic and political issues (Mann, 1987). Despite the global mobilization to end the HIV epidemic, there are remaining challenges that limit the impact of the efforts. The United Nations’ program on HIV/AIDS (UNAIDS) established the 90-90-90 strategy, aiming for 90% of people living with HIV (PLHIV) to be aware of their status, 90% of those diagnosed to initiate antiretroviral therapy (ART), and 90% of those on ART to have viral loads (VL) suppressed below levels of detection, by 2020 (UNAIDS, 2014). These goals show that viral suppression represents a key to ending the global HIV epidemic. The aim of clinical management of HIV is long-term viral suppression. Given the importance of viral suppression in HIV clinical management and epidemic control, it would be of great utility to be able to predict it among PLHIV through the continuum of care: (i) HIV diagnosis, (ii) linkage to HIV medical care, (iii) receipt of HIV medical care, (iv) retention in medical care, and (v) achievement and maintenance of viral suppression (Gardner et al., 2011). For healthcare providers and PLHIV, predicting viral suppression could help comply with treatment and possibly adjust treatment to prevent virologic failure. That is where ML could contribute to a better monitoring of patients under ART.

Several studies have been conducted to identify factors of viral suppression among PLHIV. These studies identified factors such as gender, marital status, age, added body mass index, treatment regimen, clinical stage of the infection at the time of ART initiation, duration on ART, treatment adherence, active Tuberculosis, initial fasting glucose, alcoholism, smoking, facility type, baseline CD4 count, and recent CD4 count, availability of a daily caregiver, social isolation, high stigma, and belief that there is a cure for the acquired immunodeficiency syndrome (AIDS) (Sinai et al., 2019; Ssemwanga et al., 2020; Njuguna et al., 2020; Maina et al., 2020; Hicham et al., 2019; Desta et al., 2020; Chhim et al., 2018; Rangarajan et al., 2016; Lokpo et al., 2020; Sunkanmi et al., 2020; Bulage et al., 2017). These factors can be grouped into several categories such as social and demographic, behavioral, structural and clinical factors, and provide an orientation for the choice of variables to include in a predictive model for viral load suppression.

With the growing availability of data in clinical settings, machine learning (ML) is being used for several purposes such as diagnosis, patient outcome prediction, personalized care, drug discovery, clinical trial, radiology and radiotherapy, smart electronic health records, and epidemic outbreak prediction. ML is categorized into supervised and unsupervised learning algorithms. Supervised learning algorithms are developed to predict or classify known outcomes with sets of predictors. When outcomes are unknown, unsupervised learning algorithms are used to partition samples into distinct groups where individuals of the same group have similar characteristics. Logistic regression (LR), decision trees (DT), boosted trees (BT), random forests (RF), naïve Bayes (NB), support vector machines (SVM), nearest neighbors (K-NN), and neural networks (NN) are some of the popular supervised learning algorithms (Mastoli, 2019). Classification tasks are the cornerstone of ML applications in healthcare. They can be used to predict patient outcome, diagnose, or inform treatment decisions. Given the variety of classification algorithms available, one of the challenges is to select the most suitable algorithms for healthcare datasets (Weng, 2020).

Most studies focused on using statistical analysis to identify factors of HIV care outcomes such as VL suppression (Sinai et al., 2019; Ssemwanga et al., 2020; Njuguna et al., 2020; Maina et al., 2020; Hicham et al., 2019; Desta et al., 2020; Chhim et al., 2018; Rangarajan et al., 2016; Lokpo et al., 2020; Sunkanmi et al., 2020; Bulage et al., 2017). However, HIV clinical care and research are not outside the trend of ML applications in healthcare. Although statistical analysis continues to be the prevalent application model, the use of ML is progressively expanding to encompass practical tools for clinical use to facilitate clinical decision-making (Bisaso et al., 2017).

Several studies conducted globally have utilized ML and other methodologies to predict VL. Some studies were based on HIV simulation models and others were conducted in clinical research setting (Hillmann et al., 2020; Bisaso et al., 2017; Lutz et al., 2021). Yet, research is scarce in existing literature focusing on the use of ML to assist in the treatment and management of ART programs, especially in resource-limited settings (Seboka et al., 2023), such as in Guinea. Such studies were conducted only in a couple of African countries such as Ethiopia and South Africa (Seboka et al., 2023; Maskew et al., 2022; Mamo et al., 2023).

Therefore, this study was conducted to determine the most predictive variables of VL suppression for PLVIH and develop ML models for prediction of VL suppression among PLHIV in Conakry (Guinea), using their baseline and follow-up demographic and clinical data.

## Methodology

### Study dataset

The study was conducted on a cohort of HIV patients managed in eight healthcare facilities supported by Médecins Sans Frontières (MSF), which is implementing a project aiming at the reduction of mortality and morbidity of PLHIV in Conakry (Guinea).

Disidentified patient data were extracted from the Three Interlinked Electronic Register (TIER.Net) used by the MSF’s HIV/TB Project in Conakry, Guinea (Table 1). The TIER.Net is designed with modules to capture patient-level data on HIV counseling and testing (HCT), pre-ART and ART services (Osler et al., 2014). The data set contains 20 variables with 30,205 total records, including 20,878 (69%) women, and 9,327 (31%) men. In terms of the regimen schedule, 10% (3,032) were on a 3-month schedule, 26% (7,957) on a 6-month schedule, and 64% (19,216) on a regular schedule. Prior ART, 83% (25,104) were naïve, meaning they had never received antiretroviral drugs before being included in the cohort. Regarding their method into ART, 67% (20,275) were new, and 16% (4,750) were transferred. The last pre-ART stage data showed 8% (2,539) at stage 1, 10% (2,903) at stage 2, 35% (10,662) at stage 3, and 5% (1,555) at stage 4. The variables in the dataset are mixed numerical and categorical.

**Table 1:** List of variables extracted from the TIER.Net.

Category	Variables
Demographic	
	Gender, Age At ART Start, Current Age
Clinical	
	Gender, Regimen schedule, Prior ART, Method into ART, Baseline CD4, Last Pre-ART CD4, Last ART CD4, Last Pre-ART Stage, Stage at ART Start, TB Treatment Started, TPT Outcome, Regimen At Baseline, Last ART Prescription, Second Line Start Date, TB Status At Last Visit, CPT at ART Start, Duration on ART (months), Last ART VL Count
Structural	
	Facility

Gender: The biological classification of patients as male or female; Age At ART Start: The age of the patient when they began ART; Current Age: The patient’s age at the most recent data point or clinical visit; Regimen Schedule: The specific timing and dosage plan of antiretroviral therapy (ART) medications; Prior ART: Indicates whether the patient has previously received ART before enrolling into the cohort; Method into ART: The process through which the patient was introduced to ART (new, meaning following testing or referral from another program); Baseline CD4: The CD4 cell count at the start of ART, indicating immune system strength; Last Pre-ART CD4: The CD4 count measured immediately before starting ART; Last ART CD4: The most recent CD4 count recorded while the patient is on ART; Last Pre-ART Stage: The stage of HIV progression before starting ART; Stage at ART Start: The stage of HIV progression at the time the patient began ART; TB Treatment Started: Indicates whether tuberculosis treatment was initiated; TPT Outcome: The result of tuberculosis preventive therapy; Regimen At Baseline: The initial combination of ART drugs prescribed to the patient; Last ART Prescription: The most recent ART drug prescription given to the patient; Second Line Start Date: The date when the patient began a second-line ART regimen, usually due to failure of the initial treatment; TB Status At Last Visit: The patient’s tuberculosis status during their most recent clinical visit; CPT at ART Start: Indicates whether the patient was on Cotrimoxazole prophylaxis therapy when they began ART; Duration on ART (months): The total number of months the patient has been on ART; Last ART VL Count: The most recent viral load measurement, indicating the amount of HIV in the blood; Facility: The healthcare facility where the patient receives their ART treatment, which may impact the quality of care.

### Data cleaning and pre-processing

Some variables were recoded into new ones and some records were removed for not meeting criteria (Duration on ART less than 3 months, and missing VL). These actions were taken for data cleaning at this step before further data exploration and preparation (Table 2).

**Table 2:** Data cleaning tasks performed on the original dataset.

Procedure	Variable	Task description	Rational
Variable recoding			
	VL Suppressed^a^ (Target variable)	Use Last ART VL Count to create a binary variable indicating VL suppression (Yes/No)	A threshold of <1,000 RNA copies/mL is used to define suppressed viral load (WHO, 2016)
	Second Line Treatment^a^	Use Second Line Start Date to create a binary variable indicating if patient is on second line treatment (Yes/No)	Reported date is indicative that patient is on second line treatment
	Baseline CD4, Last Pre-ART CD4 Count, and Last ART CD4 Count	Bin CD4 values into 100-unite ranges	CD4 <200 cells/μL is the threshold of immunologic failure
	Age At ART Start, Current Age	Bin ages into 5-year age groups	5 years is a common interval for age groups creation
	Duration on ART (months)	Bin Duration on ART (months) into 6-month categories	Decision is taken to categorize at each semester
Record removal			
	VL Suppressed	Remove records with missing values	VL Suppressed is the target variable. Thus, only non-missing records will be kept in the final dataset
	Duration on ART (months)	Remove records with values <3	The minimum timeline to expect viral suppression after ART initiation is 06 months (Ali and Yirtaw, 2019). Decision was made to consider 3 months after ART initiation
	Current Age	Remove records with values <18	Because of ethical considerations, under-18 patients were not included
Missing values imputation			
	TPT Outcome	Fill missing values as No treatment	Missing TPT Outcome is indicative that patient is not under TPT treatment

VL, viral load; ART, antiretroviral therapy; TPT, tuberculosis preventive treatment; CD4, count of cluster of differentiation 4 lymphocytes; TPT, tuberculosis preventive treatment.

a Newly generated variable.

The above-described processes returned a dataset of 21 variables with 13,529 records. The majority of variables have full records, but some others, namely, Baseline CD4, Last Pre-ART CD4, Last ART CD4, Last Pre-ART Stage, Stage at ART Start, TB Treatment Started, Age At ART Start, TB Status At Last Visit, CPT at ART Start, and Duration on ART have missing values ranging between 1% and 59%.

The cleaned dataset was split into training and test sets (test size = 30%), as recommended by Sidey-Gibbons and Sidey-Gibbons (2019). The following actions were taken during pre-processing: discretization of categorical variables, correction of class imbalance (88% in the positive class in the training dataset), and imputation of missing variables. Small categories were aggregated into a single category for independent variables. Specifically, categories with frequencies less than 1.50% were combined into an “Other” category to ensure sufficient sample sizes for statistical analysis and modeling (Supplementary Table S3). Additionally, nominal categorical variables were discretized by converting them into dummy variables. For the target variable, class imbalance was corrected by performing a simple bootstrapping technique which consisted of oversampling the minority category (“VL Suppressed” = No; number of samples = 8,392; random state = 5). Missing values imputation was performed with K-NN (*K* = 5) which was chosen for its capability to produce estimations close to reality and preserve the associations in the dataset (Sania et al., 2020). After the train/test split, the training and test sets were pre-processed separately to prevent information leakage from the training to the test set, and bootstrapping on the minority class was performed on the training set only (Mierswa, 2017).

The data preparation processes returned a training set and a test set of 45 numerical variables with 16,793 records in the training set (including bootstrap replicates) and 4,054 records in the test set. Understandably, VL suppression is notably higher in the test set (88.73%) compared to the training set (50.03%).

### Modeling

Eight classifiers were developed: four individual classifiers (SVM, RF, NB, and LR) and four stacked classifiers using combinations of the four classifiers (Cortes and Vapnik, 1995; Ho, 1995; Hand and Yu, 2001; Hosmer and Lemeshow, 2000; Wolpert, 1992). Linear SVM failed to converge, so only a non-linear kernel, namely, a radial basis function (RBF) kernel was used (Schlkopf and Smola, 2001). The Newton–Cholesky method was the solver used for the LR, as it is adapted to the large size of the training set and the binary classification task (Bräuninger, 1980).

The approach of 10 × 10 cross validation was used for hyperparameter tuning and for estimating the generalization performance of models. In the inner loop, a grid search was performed over a predefined range of hyperparameters for each model, to identify the optimal model configuration for individual models. This grid search systematically explored the hyperparameter space to identify the best-performing configurations. Specifically, a 10-fold cross-validation as used to evaluate each combination of hyperparameters within the grid (Claesen and De Moor, 2015). In the grid search the penalty C and gamma parameters were used for SVM, the number of estimators and maximum depth were used for RF, and the maximum iteration parameter was used for LR. NB, as a probabilistic classifier with no hyperparameters, it was not subject to grid search (Table 3).

**Table 3:** Summary of parameter optimization on balanced subset.

Algorithm	Default parameters	Parameters optimized and values considered	Best parameters	Mean accuracy (95% CI)
SVM	Kernel = RBF Random state = 40	C: 10, 20, 30, 40, 50, 60, 70, 80, 90, 100 Gamma: 0.5, 0.6, 0.7, 0.8, 0.9	*C* = 10 Gamma = 0.9	0.75 (0.74–0.77)
RF	Random state = 40	Number of estimators: 500, 1,000, 1,500, 2000 Maximum depth: None, 10, 30, 50, 70, 90, 100	Number of estimators = 1,000 Maximum depth = None	0.82 (0.80–0.83)
NB	Not applicable	Not applicable	None	0.72 (0.70–0.76)
LR	Random state = 40 Solver = Newton–Cholesky	Maximum iteration: 100, 200, 300, 400, 500, 600, 700, 800, 900	Maximum iteration = 100	0.75 (0.74–0.77)

In the outer loop, and following identification of the best model configuration from the grid search, a second round of 10-fold cross-validation was conducted on a 10% random subset of the training data (1,679 samples) to assess the variance of the selected model (Cawley and Talbot, 2010; Stone, 1974). The random state was set to 40 to ensure reproducibility of the results. Mean accuracies for each model configuration were calculated, along with their 95% confidence intervals (95% CI) (Table 3). Finally, the best performing models were applied to the unbalanced test dataset (*n* = 4,054) to estimate accuracy on unseen data.

After developing the SVM, RF, NB and LR algorithms, they were input into four other stacked classifiers, aiming at leveraging the performance of the individual classifiers. The output of three individual classifiers were stacked as inputs (classifiers) to the fourth one used as classifier to compute the final prediction (meta classifier): [inputs = (LR, NB, RF), meta classifier = SVM]; [inputs = (LR, NB, SVM), meta classifier = RF]; [inputs = (LR, SVM, RF), meta classifier = NB]; [inputs = (NB, SVM, RF), meta classifier = LR].

RF was used to determine the variables importance in predicting VL suppression. The feature importance attribute was fitted on the RF classifier to determine the most predictive variables of VL suppression. A summary plot of the variables, ranked by their importance scores displayed the importance of each variable based on the RF model.

### Evaluation

The performance of each of the four individual algorithms and the four stacked algorithms was measured on the test set using sensitivity, specificity, predictive positive value (PPV), predictive negative value (PNV), *F*-score, and area under the curve (AUC) as evaluation metrics (Rainio et al., 2024). The *F*-score combines positive predictive value (precision) with sensitivity and is a relevant metric to assess the models’ capability to predict the target positive class (VL Suppressed = 1) (Christen et al., 2023). In clinical practice, predicting suppressed viral load is equally important as predicting non-suppressed viral load. Thus, in addition to *F*-score, AUC, which also considers the negative class (VL Suppressed = 0), was considered in the models’ evaluation (Fawcett, 2006). Determining the best performing model consisted in finding the optimal balance between *F*-score and AUC.

**Figure 1:**
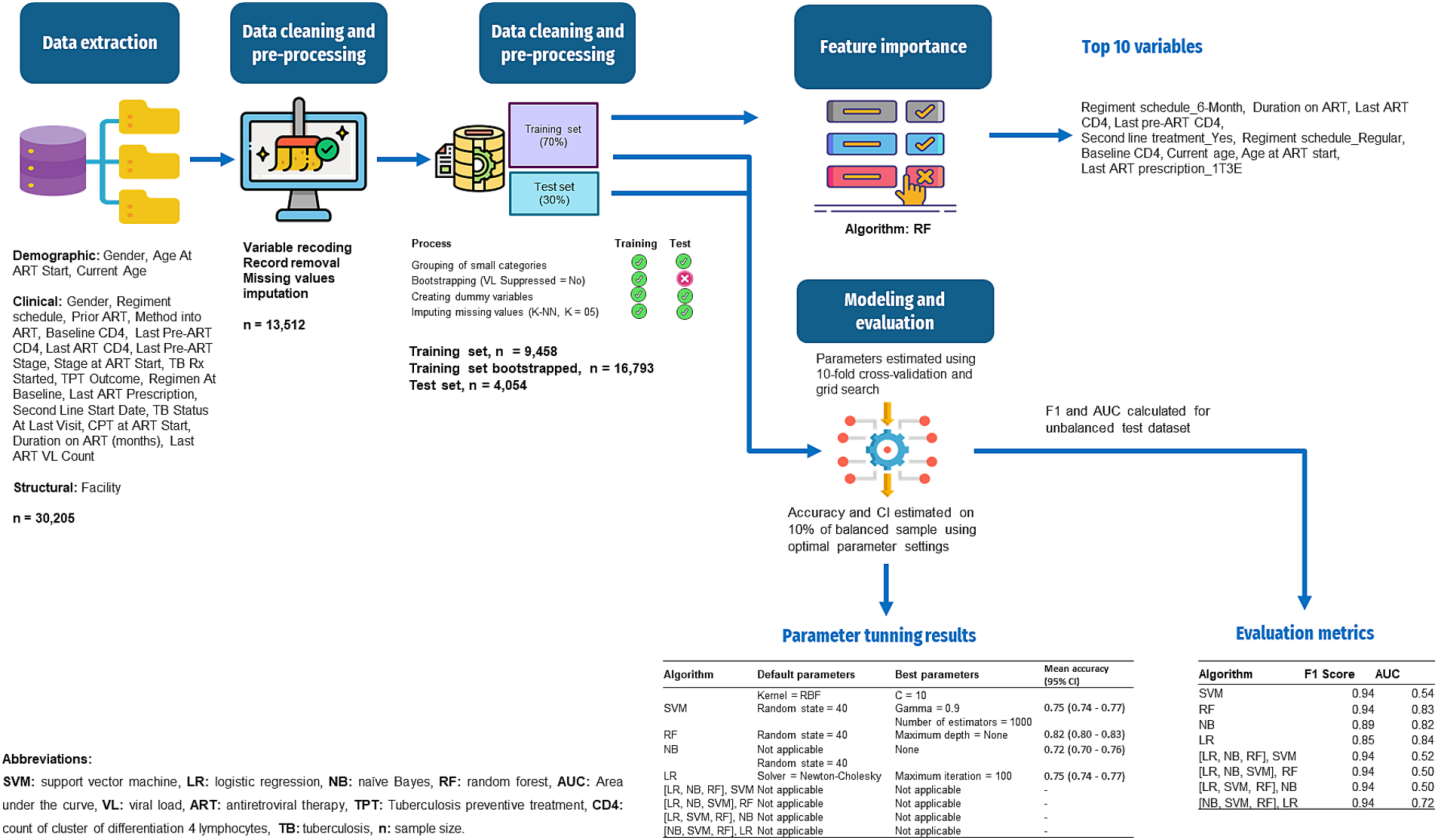
Workflow of the ML for prediction of VL suppression among HIV patients in Conakry, Guinea.

In summary, the ML pipeline developed for this study is presented in Figure 1.

## Results

**Figure 2:**
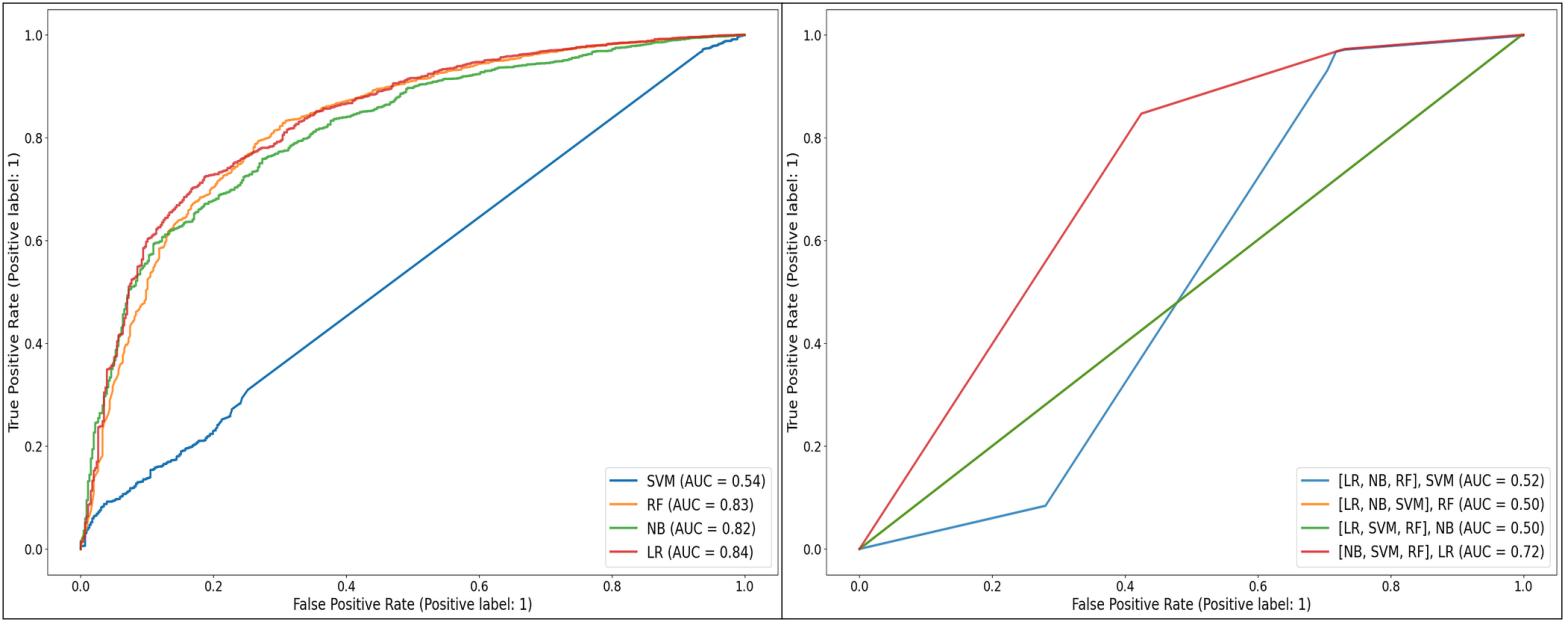
Receiver operating characteristic curves of the individual and stacked models developed, when applied to the unbalanced test dataset.

The RF’s feature importance revealed that, Regimen schedule_6-Month, Duration on ART (months), Last ART CD4, Regimen schedule_Regular, Last Pre-ART CD4, Second Line Treatment_Yes, Baseline CD4, Current Age, Age At ART Start, and Last ART Prescription_1T3E were the top 10 most predictive variables for VL suppression. The complete overview of feature importance based on the RF model is presented on Figure 2.

As depicted in Table 3, SVM was initially set with an RBF kernel and a random state of 40. From the “*C*” parameter values in the range of 10 to 100 and gamma values from 0.5 to 0.9 that were optimized, the best performance was achieved with *C* = 10 and gamma = 0.9, yielding a mean accuracy of 0.75 [95% CI (0.74–0.77)] on the balanced training dataset. RF was initially set with a random state of 40 and optimized over the number of estimators ranging from 500 to 2000, and the maximum tree depth, with options of None or values from 10 to 100. The optimal model was found with 1,000 estimators and no limit on the maximum depth, achieving a mean accuracy of 0.82 [95% CI (0.80–0.83)] on the balanced training dataset. NB, which does not have applicable parameters to optimize, achieved a mean accuracy of 0.72 [95% CI (0.70–0.76)] on the balanced training dataset. Lastly, LR, initially set with a random state of 40 and using the Newton–Cholesky solver, was optimized over the maximum number of iterations, ranging from 100 to 900. The optimal model was found with a maximum of 100 iterations, resulting in a mean accuracy of 0.75 [95% CI (0.74–0.77)] on the balanced training dataset.

The models’ evaluation metrics showed that SVM, RF, [(LR, NB, RF), SVM], [(LR, NB, SVM), RF], [(LR, SVM, RF), NB], and [(NB, SVM, RF), LR] performed highly based on *F*-score for the positive class (94%) when applied to the unbalanced test dataset. NB and LR performed lower with *F*-scores of 89% and 85%, respectively (Table 4).

**Table 4:** Summary confusion matrixes and evaluation metrics of the individual and stacked models developed, when applied to the unbalanced test dataset.

Model	Predicted positive	Predicted negative	Sensitivity	Specificity	PPV	PNV	*F*-score
SVM							
Actual positive	3,597	0	1	0	0.89	1	0.94
Actual negative	456	1	—	—	—	—	—
RF^a^							
Actual positive	3,480	117	0.97	0.28	0.91	0.52	0.94
Actual negative	328	129	—	—	—	—	—
NB^a^							
Actual positive	3,064	533	0.85	0.56	0.94	0.33	0.89
Actual negative	200	257	—	—	—	—	—
LR							
Actual positive	2,748	849	0.76	0.75	0.96	0.29	0.85
Actual negative	115	342	—	—	—	—	—
(LR, NB, RF), SVM							
Actual positive	3,480	117	0.97	0.28	0.91	0.52	0.94
Actual negative	328	129	—	—	—	—	—
(LR, NB, SVM), RF							
Actual positive	3,597	0	1	0	0.89	1	0.94
Actual negative	456	1	—	—	—	—	—
(LR, SVM, RF), NB							
Actual positive	3,597	0	1	0	0.89	1	0.94
Actual negative	456	1	—	—	—	—	—
(NB, SVM, RF), LR							
Actual positive	3,498	99	0.97	0.27	0.91	0.55	0.94
Actual negative	334	123	—	—	—	—	—

PPV, predictive positive value; PNV, predictive negative value.

a Algorithms with the optimal balance between *F*-score and AUC.

**Figure 3:**
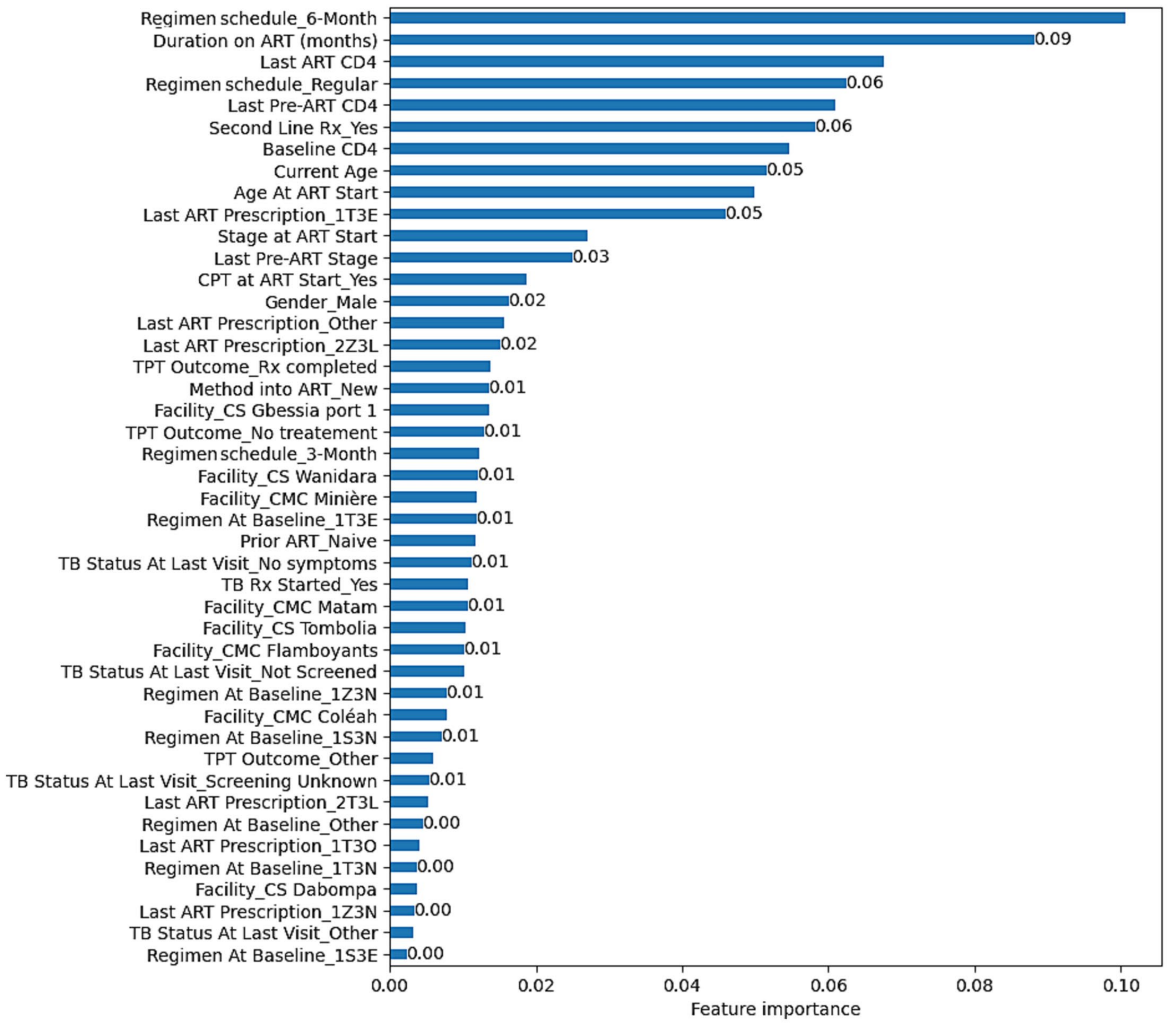
Feature importance based on the RF model developed to predict VL suppression among HIV patients in Conakry, Guinea.

Unsurprisingly, given the level of class imbalance, AUC scores did not concur. SVM, [(LR, NB, RF), SVM], [(LR, NB, SVM), RF], and [(LR, SVM, RF), NB] showed poor performances—not better than random guesses, with AUCs between 50% and 54%, while [(NB, SVM, RF), LR] performed moderately with 73% AUC (Figure 3).

RF (82% AUC), NB (82% AUC), and LR (84% AUC) stood out with the highest AUCs. Considering additional metrics, RF yielded higher sensitivity (97%) and *F*-score (94%), highlighting its strength in correctly classifying positive classes and achieving a balanced precision-recall performance. However, its specificity was low at 28%. NB model showed a moderate sensitivity (85%) and a higher specificity (56%) relative to RF. Although LR showed the highest specificity (75%), its sensitivity was the lowest (76%) (Table 4).

Aiming at finding the best balance between the capability to predict the positive class (VL suppression) as well as predicting the negative class (VL non-suppression), AUC has been weighted in for its indication in discriminating between the target classes. Moreover, in a cohort where the non-suppressed VL is the minority class, it is critical to select a model that can detect it, meaning a model with a high specificity. Looking for the best balance between *F*-score and AUC, RF (94% *F*-score, 82% AUC) and NB (89% *F*-score, 82% AUC) are the algorithms that are optimal for predicting both classes. As illustrated in Table 3, RF had a lower model variance and a higher accuracy than NB when applied to the balanced dataset.

## Discussion

The performances produced by the NB (AUC = 82%) and RF (AUC = 82%) models developed in this study are comparable to those obtained in other studies, where the AUCs varied between 63% and 83% (Revell et al., 2012; Revell et al., 2013; Petersen et al., 2015; Kamal et al., 2021). For example, Revell et al. (2012) developed a RF model to predict VL reduction using data from North America, Western Europe and Australia. After excluding the genotype variable, implementing a model improvement strategy, and testing on data from Romania, the model produced an AUC equal to 83%. Revell et al. (2013) also developed models that can predict VL suppression without a genotype and evaluated their applicability in resource-limited settings. The models were trained using data from well-resourced countries and evaluated data from well-resourced countries mixed with data from Southern Africa, India, and Romania. The models achieved an AUC of 76%–77% with the test samples from well-resourced countries, 58%–65% with samples from Southern Africa, 63% with samples from India, and 70% with samples from Romania.

Petersen et al. (2015) used data from US cohorts and applied a super learner algorithm for classifying virologic failure. The results showed that AUC was 78 and 79% for virologic failures at >1,000 copies/mL or >400 copies/mL thresholds, respectively. Kamal et al. (2021) developed a RF to predict viral rebound from medication adherence and clinical data in Switzerland, which produced an average AUC of 65%. It can be observed that some of these models performed poorer (63%), while the top performing yielded exactly 83% AUC as in this project. Models tested by Revell et al. (2013) in different contexts produced lower performances as compared to testing with dataset from the setting where the training sets were collected.

A couple of similar studies have been conducted in Africa. Maskew et al. (2022) applied ML to viral suppression in South African HIV treatment cohorts and obtained a performance of 76% AUC. Mamo et al. (2023) developed various ML algorithms for predicting virological failure using HIV treatment cohort data from Gondar Comprehensive and Specialized Hospital in Ethiopia. Among these algorithms, the RF outperformed with nearly 100% AUC (0.9989), although with a smaller sample size and just 141 instances in the minority class. Seboka et al. (2023) also developed several algorithms with data from HIV treatment cohorts from Gedeo Zone Public Hospitals in Ethiopia and found that eXtreme Gradient Boosting (XGB) and RF performed as the best algorithms for viral load prediction with 99% AUC, also with a small sample size (minority class: *n* = 140).

The input variables used for these models are included in this project with additional variables such as age, tuberculosis prevention and treatment, and health facility. The variables used in the study are also in line with those found in studies conducted to identify factors of viral load suppression, with the exception of treatment adherence, marital status, initial fasting glucose, alcoholism, smoking, availability of a daily caregiver, belief that there is a cure for AIDS, social isolation, high stigma, and body mass index (Sinai et al., 2019; Ssemwanga et al., 2020; Njuguna et al., 2020; Maina et al., 2020; Hicham et al., 2019; Desta et al., 2020; Chhim et al., 2018; Rangarajan et al., 2016; Lokpo et al., 2020; Sunkanmi et al., 2020; Bulage et al., 2017). HIV genotype was not available in the dataset, but Revell et al. (2012) demonstrated that it is possible to develop models without it and obtain results that perform as those developed with it.

The results of this study have some limitations. The data used for model building were collected in the specific context of Conakry (Guinea). It has been demonstrated that performance may be reduced while testing in a different context, and consequently, results in this study may not be maintained if the models are evaluated in different settings (Revell et al., 2013). Exploring the applicability of the results in different settings would be a relevant inquiry. Moreover, the results are dependent on the quality of the data used. The missing data imputation applied may have induced increased performance estimates of the models. TU Dublin ethical clearance was contingent on binning numeric attributes to ensure a greater level of anonymity, thus further reducing the information quality of the data. The LR chosen in this study may also be affected by its specific limitations: it requires independence of observations, absence of multicollinearity among the independent variables, and linearity of independent variables and log odds, which cannot be assured with clinical data.

This study is limited to exploring ML algorithms with the most predictive variables, the optimal parameters, and the top performing model. The development phase of the CRISP-DM methodology was not addressed in this study, and thus the results are not readily usable in clinical practice.

## Conclusion

SVM, RF, NB, LR and four stacked classifiers using combinations of the four individual ones were developed. Their evaluation showed that SVM, RF, [(LR, NB, RF), SVM], [(LR, NB, SVM), RF], [(LR, SVM, RF), NB], [(NB, SVM, RF), LR] yielded high performances based on *F*-score for the positive class (94%). When weighting in AUC, RF (94% *F*-score, 83% AUC) and NB (89% *F*-score, 82% AUC) presented the optimal balance between *F*-scores and AUCs. This means that the RF and NB are the top performing algorithms in a way that they can be used to detect both VL suppression and non-suppression. With these models, the proportions of suppressed VL and non-suppressed VL that can be detected are 97% and 28% for RF, and 85% and 56% for NB.

The optimal parameters found were *C* = 10 and gamma = 0.9 for SVM (Kernel = RBF, Random state = 40), Number of estimators = 1,000 and no maximum depth for RF (Random state = 40), Maximum iteration = 100 for LR (Random state = 40, Solver = Newton–Cholesky), and the default parameters were maintained for NB. With RF, Regimen schedule_6-Month, Duration on ART (months), Last ART CD4, Regimen schedule_Regular, Last Pre-ART CD4, Second Line Treatment_Yes, Baseline CD4, Current Age, Age At ART Start, and Last ART Prescription_1T3E were identified as the top predicting variables associated with VL suppression.

The possible future direction of this study is evaluating the models on data from different contexts to assess generalizability. Moreover, as RF and NB are the most relevant models, they can be developed into an application that can be tested in a clinical context.

## Data Availability

The data analyzed in this study is subject to the following licenses/restrictions: The data that support the findings of this study are not publicly available due to concerns regarding participant anonymity but accessible on request from Médecins Sans Frontières. Requests to access these datasets should be directed to Médecins Sans Frontières Belgium, Rue de l’Arbre Bénit 46, 1050 Bruxelles, Belgium. E-mail: dpo@brussels.msf.org. The codes developed for data cleaning, model development and evaluation are available in the Zenodo repository: https://doi.org/10.5281/zenodo.8151170.
