# Supplementary material for: Development of machine learning algorithms to predict viral load suppression among HIV patients in Conakry (Guinea)
Source: Front Artif Intell. 2025 Mar 19;8:1446876. doi: 10.3389/frai.2025.1446876 (PMC11961888; doi:10.3389/frai.2025.1446876)
Supplement: Supplementary file 1 [file Data_Sheet_1.docx]

**Glossary**

**Regimen Schedule:** The specific timing and dosage plan of antiretroviral therapy (ART) medications that a patient follows. This schedule is crucial for ensuring the effectiveness of the treatment in controlling HIV.

**Prior ART:** Indicates whether a patient has received ART before enrolling in the cohort. Patients who are "ART naive" have never taken ART, while those with prior ART experience have previously undergone treatment.

**Method into ART:** The way in which a patient was introduced to ART, which could include starting treatment due to a diagnosis of HIV, or a referral from another program.

**Baseline CD4:** The CD4 count – a type of white blood cell that is crucial for immune function, measured at the start of ART. A higher CD4 count generally indicates a stronger immune system.

**Last Pre-ART CD4:** The CD4 count measured immediately before a patient begins ART. This provides a snapshot of the patient’s immune function before starting treatment.

**Last ART CD4:** The most recent CD4 count measured while the patient is on ART. This helps monitor how well the patient is responding to treatment.

**Last Pre-ART Stage:** The stage of HIV disease progression (e.g., Stage 1 to 4) that a patient was in before starting ART. This staging helps assess the severity of the disease.

**Stage at ART Start:** The stage of HIV disease progression at the time when the patient began ART. Understanding this helps in evaluating the urgency and intensity of the treatment required.

**TB Rx Started:** Indicates whether treatment for tuberculosis (TB) was started, as HIV patients are at higher risk for developing TB, which can complicate their treatment and care.

**TPT Outcome:** The outcome of TB preventive therapy (TPT), which is given to prevent the development of active TB in patients with HIV.

**Age at ART Start:** The age of the patient when they began ART. This can impact how they respond to treatment and the types of side effects they might experience.

**Regimen at Baseline:** The specific combination of antiretroviral drugs that a patient was initially prescribed when they started ART. Different regimens may be chosen based on patient factors and drug availability.

**Last ART Prescription:** The most recent prescription of antiretroviral drugs given to the patient. Monitoring changes in prescriptions helps in understanding treatment adjustments or responses to side effects.

**Facility:** The healthcare facility where the patient receives their treatment. This can impact the quality of care, access to medications, and overall health outcomes.

**TB Status at Last Visit:** The patient’s TB status during their last visit, indicating whether they are currently being treated for TB, have been cured, or have no history of TB.

**CPT at ART Start:** Refers to the use of Cotrimoxazole prophylaxis therapy (CPT), a medication used to prevent certain infections in HIV patients, at the start of ART.

**Duration on ART (months):** The total amount of time, in months, that the patient has been on antiretroviral therapy. This helps assess the long-term effects and sustainability of the treatment.

**Second Line Rx:** Indicates whether the patient has been switched to a second-line ART, typically used when the first regimen fails due to drug resistance or side effects.

**VL Suppressed:**

Refers to whether the patient’s viral load (amount of HIV in the blood) is below detectable levels due to effective ART. Viral load suppression is the goal of HIV treatment, indicating that the virus is under control.

**Table S1.** Description of the original dataset extracted from the TIER.Net

| **#** | **Variables** | **Data type** | **Number of records** | **% Missing records** | **Class (n, %)** |
| --- | --- | --- | --- | --- | --- |
| 1 | Gender | object | 30205 | 0 | Female (20878, 69%), Male (9327, 31%) |
| 2 | Regimen schedule | object | 30205 | 0 | 3-Month (3032, 10%), 6-Month (7957, 26%), Regular (19216, 64%) |
| 3 | Prior ART | object | 28725 | 5 | Missing (1480, 5%), Naive (25104, 83%), Non-naive (3621, 12%) |
| 4 | Method into ART | object | 25025 | 17 | Missing (5180, 17%), New (20275, 67%), Transferred (4750, 16%) |
| 8 | Last Pre-ART Stage | float64 | 17659 | 42 | Missing (12546, 42%), 1 (2539, 8%), 2 (2903, 10%), 3 (10662, 35%), 4 (1555, 5%) |
| 9 | Stage at ART Start | float64 | 24590 | 19 | Missing (5615, 19%), 1 (5520, 18%), 2 (3319, 11%), 3 (13883, 46%), 4 (1868, 6%) |
| 10 | TB Rx Started | object | 22612 | 25 | Missing (7593, 25%), No (19152, 63%), Yes (3460, 11%) |
| 11 | TPT Outcome | object | 10669 | 65 | Missing (19536, 65%), Developed TB (20, 0%), Rx completed (9938, 33%), Rx interrupted (697, 2%), Transferred (14, 0%) |
| 14 | Regimen At Baseline | object | 25038 | 17 | Missing (5167, 17%), 1A3E (382, 1%), 1A3L (357, 1%), 1A3N (9, 0%), 1A3O (6, 0%), 1A3R (1, 0%), 1A3V (1, 0%), 1S3 (5, 0%), 1S3A (3, 0%), 1S3E (437, 1%), 1S3L (99, 0%), 1S3N (3520, 12%), 1T3 (3, 0%), 1T3E (15759, 52%), 1T3L (86, 0%), 1T3N (587, 2%), 1T3O (392, 1%), 1T3V (15, 0%), 1TFE (136, 0%), 1Z3 (2, 0%), 1Z3A (5, 0%), 1Z3E (362, 1%), 1Z3L (50, 0%), 1Z3LT (2, 0%), 1Z3N (2748, 9%), 1Z3O (2, 0%), 1Z3V (1, 0%), 1ZE (1, 0%), 1ZN (1, 0%), 2A3L (18, 0%), 2A3O (1, 0%), 2T3L (14, 0%), 2T3O (8, 0%), 2T3V (1, 0%), 2Z3L (17, 0%), 2Z3O (1, 0%), 2Z3V (3, 0%), 3A3O (1, 0%), STOPPED (2, 0%) |
| 15 | Last ART Prescription | object | 25058 | 17 | Missing (5147, 17%), 1A3E (424, 1%), 1A3L (314, 1%), 1A3N (19, 0%), 1A3O (10, 0%), 1A3V (5, 0%), 1S3E (172, 1%), 1S3L (29, 0%), 1S3N (962, 3%), 1T3E (17843, 59%), 1T3EO (42, 0%), 1T3L (55, 0%), 1T3N (199, 1%), 1T3NO (1, 0%), 1T3O (1131, 4%), 1T3V (22, 0%), 1TFE (46, 0%), 1Z3A (1, 0%), 1Z3E (324, 1%), 1Z3EO (1, 0%), 1Z3L (61, 0%), 1Z3LT (1, 0%), 1Z3N (1525, 5%), 1Z3NT (1, 0%), 1Z3O (12, 0%), 1Z3V (5, 0%), 2A3E (9, 0%), 2A3L (294, 1%), 2A3LT (2, 0%), 2A3O (18, 0%), 2A3V (62, 0%), 2AdL (1, 0%), 2T3E (7, 0%), 2T3L (389, 1%), 2T3LA (1, 0%), 2T3LO (1, 0%), 2T3LT (1, 0%), 2T3LZ (1, 0%), 2T3N (1, 0%), 2T3O (166, 1%), 2T3V (124, 0%), 2T3VA (1, 0%), 2TFE (1, 0%), 2Z3E (8, 0%), 2Z3L (525, 2%), 2Z3LT (3, 0%), 2Z3N (2, 0%), 2Z3O (68, 0%), 2Z3OT (2, 0%), 2Z3V (109, 0%), 2Z3VT (3, 0%), 2ZdN (1, 0%), 3A3O (4, 0%), 3A3V (1, 0%), 3T3E (1, 0%), 3T3O (38, 0%), 3T3OL (1, 0%), 3T3OU (1, 0%), 3T3VO (1, 0%), 3T3Z (1, 0%), 3Z3L (1, 0%), 3Z3LO (1, 0%), 3Z3O (3, 0%) |
| 17 | Facility | object | 30205 | 0 | CMC Matam (10054, 33%), CS Gbessia port 1 (4274, 14%), CMC Flamboyants (3336, 11%), CS Tombolia (3306, 11%), CS Wanidara (3283, 11%), CMC Minière (3088, 10%), CMC Coléah (1850, 6%), CS Dabompa (1014, 3%) |
| 18 | TB Status At Last Visit | object | 24596 | 19 | Missing (5609, 19%), No symptoms (15707, 52%), Not Screened (4812, 16%), Screening Unknown (2782, 9%), On TB Rx (1216, 4%), Symptoms (79, 0%) |
| 19 | CPT at ART Start | object | 17786 | 41 | Missing (12419, 41%), Oui (13170, 44%), Non (4616, 15%) |
| 5 | Baseline CD4 | float64 | 16102 | 47 | Mean: 298 Std: 266 Min: 0.0047 Max: 3000 |
| 6 | Last Pre-ART CD4 | float64 | 10287 | 66 | Mean: 276 Std: 250 Min: 0 Max: 3000 |
| 7 | Last ART CD4 | float64 | 18813 | 38 | Mean: 449 Std: 306 Min: 0.03 Max: 2992 |
| 12 | Age At ART Start | float64 | 25058 | 17 | Mean: 3 Std: 1 Min: 1 Max: 4 |
| 13 | Current Age | int64 | 30205 | 0 | Mean: 33 Std: 13 Min: 0 Max: 90 |
| 16 | Second Line Start Date | datetime64[ns] | 1939 | 94 | Oldest date: 4/16/2007, Earliest date: 8/31/2021, Most frequent date: 5/26/2017 |
| 20 | Duration on ART (months) | float64 | 29430 | 3 | Mean: 49 Std: 48 Min: 0 Max: 246 |
| 21 | Last ART VL Count | float64 | 14252 | 53 | Mean: 34025 Std: 277679 Min: 0 Max: 9147452 |

Table S2. Characteristics of the initial training and test sets

| **#** | **Variable** | **Data type** | **Training set** | |  | **Test set** | |
| --- | --- | --- | --- | --- | --- | --- | --- |
|  |  |  | **Number of records** | **% Missing values** |  | **Number of records** | **% Missing values** |
| 0 | Gender | object | 9470 | 0 |  | 4059 | 0 |
| 1 | Regimen schedule | object | 9470 | 0 |  | 4059 | 0 |
| 2 | Prior ART | object | 9470 | 0 |  | 4059 | 0 |
| 3 | Method into ART | object | 9470 | 0 |  | 4059 | 0 |
| 4 | Baseline CD4 | category | 6232 | 34 |  | 2583 | 36 |
| 5 | Last Pre-ART CD4 | category | 3911 | 59 |  | 1612 | 60 |
| 6 | Last ART CD4 | category | 7812 | 18 |  | 3336 | 18 |
| 7 | Last Pre-ART Stage | float64 | 4872 | 49 |  | 2030 | 50 |
| 8 | Stage at ART Start | float64 | 9399 | 1 |  | 4028 | 1 |
| 9 | TB Rx Started | object | 8561 | 10 |  | 3658 | 10 |
| 10 | TPT Outcome | object | 9470 | 0 |  | 4059 | 0 |
| 11 | Age At ART Start | category | 9362 | 1 |  | 4007 | 1 |
| 12 | Current Age | category | 9470 | 0 |  | 4059 | 0 |
| 13 | Regimen At Baseline | object | 9468 | 0 |  | 4059 | 0 |
| 14 | Last ART Prescription | object | 9470 | 0 |  | 4059 | 0 |
| 15 | Facility | object | 9470 | 0 |  | 4059 | 0 |
| 16 | TB Status At Last Visit | object | 9408 | 1 |  | 4033 | 1 |
| 17 | CPT at ART Start | object | 6829 | 28 |  | 2889 | 29 |
| 18 | Duration on ART (months) | category | 8561 | 10 |  | 3642 | 10 |
| 19 | Second Line Rx | object | 9470 | 0 |  | 4059 | 0 |
| 20 | VL Suppressed | object | 9470 | 0 |  | 4059 | 0 |

Table S3. Description of the pre-processing technics performed on independent and target variables

| **Variable** | | **Description of the process** |
| --- | --- | --- |
| **Independent variable** | | **Grouping small categories** |
|  | Last ART CD4, Last Pre-ART CD4, Baseline CD4 | Keep CD4 equals 100s, 200s, 300s, 400s, and 500s as is.  Recode any CD4 equal or above 600s as 600s. |
|  | TPT Outcome | Keep Rx completed, and No treatment as is.  Recode Rx interrupted, Patient transferred, and Developed TB as Other. |
|  | Regimen At Baseline | Keep 1T3E, 1S3N, 1Z3N, 1T3N, and 1S3E as is.  Recode any other regimen as Other. |
|  | Last ART Prescription Code | Keep 1T3E, 1T3O, 2Z3L, 1Z3N, and 2T3L as is.  Recode any other regimen as Other regimen. |
|  | TB Status At Last Visit | Recode No symptoms, Not Screened, and Screening Unknown as is.  Recode On TB treatment and Symptoms as Other. |
|  | Age At ART Start, and Current Age | Keepp age equals 20s, 25s, 30s, 35s, 40s, 45s, 50s, and 55s as is.  Recode any age equal or above 60s as 60s. |
| **Target variable** | | **Bootstrapping** |
|  | VL Suppressed_Yes | Minority class: 'VL Suppressed_Yes' = 0; number of samples= 8392; random state = 05 |

Table S4. Characteristics of the final training and test sets

| **#** | **Variable** | **Data type** | **Number of records** | |
| --- | --- | --- | --- | --- |
|  |  |  | **Training set** | **Test set** |
| 1 | Gender_Male | int64 | 16793 | 4054 |
| 2 | Regimen schedule_3-Month | int64 | 16793 | 4054 |
| 3 | Regimen schedule_6-Month | int64 | 16793 | 4054 |
| 4 | Regimen schedule_Regular | int64 | 16793 | 4054 |
| 5 | Prior ART_Naive | int64 | 16793 | 4054 |
| 6 | Method into ART_New | int64 | 16793 | 4054 |
| 7 | TB Rx Started_Yes | int64 | 16793 | 4054 |
| 8 | TPT Outcome_No treatement | int64 | 16793 | 4054 |
| 9 | TPT Outcome_Other | int64 | 16793 | 4054 |
| 10 | TPT Outcome_Rx completed | int64 | 16793 | 4054 |
| 11 | Regimen At Baseline_1S3E | int64 | 16793 | 4054 |
| 12 | Regimen At Baseline_1S3N | int64 | 16793 | 4054 |
| 13 | Regimen At Baseline_1T3E | int64 | 16793 | 4054 |
| 14 | Regimen At Baseline_1T3N | int64 | 16793 | 4054 |
| 15 | Regimen At Baseline_1Z3N | int64 | 16793 | 4054 |
| 16 | Regimen At Baseline_Other | int64 | 16793 | 4054 |
| 17 | Last ART Prescription_1T3E | int64 | 16793 | 4054 |
| 18 | Last ART Prescription_1T3O | int64 | 16793 | 4054 |
| 19 | Last ART Prescription_1Z3N | int64 | 16793 | 4054 |
| 20 | Last ART Prescription_2T3L | int64 | 16793 | 4054 |
| 21 | Last ART Prescription_2Z3L | int64 | 16793 | 4054 |
| 22 | Last ART Prescription_Other | int64 | 16793 | 4054 |
| 23 | Facility_CMC Coléah | int64 | 16793 | 4054 |
| 24 | Facility_CMC Flamboyants | int64 | 16793 | 4054 |
| 25 | Facility_CMC Matam | int64 | 16793 | 4054 |
| 26 | Facility_CMC Minière | int64 | 16793 | 4054 |
| 27 | Facility_CS Dabompa | int64 | 16793 | 4054 |
| 28 | Facility_CS Gbessia port 1 | int64 | 16793 | 4054 |
| 29 | Facility_CS Tombolia | int64 | 16793 | 4054 |
| 30 | Facility_CS Wanidara | int64 | 16793 | 4054 |
| 31 | TB Status At Last Visit_No symptoms | int64 | 16793 | 4054 |
| 32 | TB Status At Last Visit_Not Screened | int64 | 16793 | 4054 |
| 33 | TB Status At Last Visit_Other | int64 | 16793 | 4054 |
| 34 | TB Status At Last Visit_Screening Unknown | int64 | 16793 | 4054 |
| 35 | CPT at ART Start_Yes | int64 | 16793 | 4054 |
| 36 | Second Line Rx_Yes | int64 | 16793 | 4054 |
| 37 | Baseline CD4 | int64 | 16793 | 4054 |
| 38 | Last Pre-ART CD4 | int64 | 16793 | 4054 |
| 39 | Last ART CD4 | int64 | 16793 | 4054 |
| 40 | Last Pre-ART Stage | int64 | 16793 | 4054 |
| 41 | Stage at ART Start | int64 | 16793 | 4054 |
| 42 | Age At ART Start | int64 | 16793 | 4054 |
| 43 | Current Age | int64 | 16793 | 4054 |
| 44 | Duration on ART (months) | int64 | 16793 | 4054 |
| 45 | VL Suppressed_Yes | int64 | 16793 | 4054 |

**Table S5.** Frequency distributions of the final train and test sets

| **Variable** | | **Train set** |  |  | **Test set** |  |
| --- | --- | --- | --- | --- | --- | --- |
|  |  | **n=16,793** | **(%)** |  | **n=4,054** | **(%)** |
| Gender (Male) | | 4,561 | 27.16 |  | 1,153 | 28.44 |
| Regimen schedule | |  |  |  |  |  |
|  | 3-Month | 2,395 | 14.26 |  | 465 | 11.47 |
|  | 6-Month | 6,023 | 35.87 |  | 2,220 | 54.76 |
|  | Regular | 8,375 | 49.87 |  | 1,369 | 33.77 |
| Prior ART_Naive | | 14,148 | 84.25 |  | 3,409 | 84.09 |
| Method into ART_New | | 12,154 | 72.38 |  | 2,803 | 69.14 |
| TB Rx Started_Yes | | 2,254 | 13.42 |  | 507 | 12.51 |
| TPT Outcome | |  |  |  |  |  |
|  | No treatement | 6,385 | 38.02 |  | 1,448 | 35.72 |
|  | Other | 716 | 4.26 |  | 135 | 3.33 |
|  | Rx completed | 9,692 | 57.71 |  | 2,471 | 60.95 |
| Regimen At Baseline | |  |  |  |  |  |
|  | 1S3E | 318 | 1.89 |  | 79 | 1.95 |
|  | 1S3N | 2,471 | 14.71 |  | 620 | 15.29 |
|  | 1T3E | 10,858 | 64.66 |  | 2,594 | 63.99 |
|  | 1T3N | 458 | 2.73 |  | 101 | 2.49 |
|  | 1Z3N | 2,081 | 12.39 |  | 501 | 12.36 |
|  | Other | 607 | 3.61 |  | 159 | 3.92 |
| Last ART Prescription | |  |  |  |  |  |
|  | 1T3E | 10,808 | 64.36 |  | 3,284 | 81.01 |
|  | 1T3O | 586 | 3.49 |  | 149 | 3.68 |
|  | 1Z3N | 583 | 3.47 |  | 99 | 2.44 |
|  | 2T3L | 883 | 5.26 |  | 96 | 2.37 |
|  | 2Z3L | 1,497 | 8.91 |  | 138 | 3.4 |
|  | Other | 2,436 | 14.51 |  | 288 | 7.1 |
| Facility | |  |  |  |  |  |
|  | CMC Coléah | 1,405 | 8.37 |  | 319 | 7.87 |
|  | CMC Flamboyants | 2,100 | 12.51 |  | 604 | 14.9 |
|  | CMC Matam | 3,058 | 18.21 |  | 619 | 15.27 |
|  | CMC Minière | 2,294 | 13.66 |  | 537 | 13.25 |
|  | CS Dabompa | 540 | 3.22 |  | 132 | 3.26 |
|  | CS Gbessia port 1 | 3,236 | 19.27 |  | 702 | 17.32 |
|  | CS Tombolia | 2,229 | 13.27 |  | 571 | 14.08 |
|  | CS Wanidara | 1,931 | 11.5 |  | 570 | 14.06 |
| TB Status At Last Visit | |  |  |  |  |  |
|  | No symptoms | 12,461 | 74.2 |  | 3,116 | 76.86 |
|  | Not Screened | 2,711 | 16.14 |  | 614 | 15.15 |
|  | Other | 532 | 3.17 |  | 75 | 1.85 |
|  | Screening Unknown | 1,089 | 6.48 |  | 249 | 6.14 |
| CPT at ART Start_Yes | | 8,827 | 52.56 |  | 2,207 | 54.44 |
| Second Line Rx | | 4,469 | 26.61 |  | 459 | 11.32 |
| VL Suppressed | | 8,401 | 50.03 |  | 3,597 | 88.73 |
| Last Pre-ART Stage | |  |  |  |  |  |
|  | Stage 1 | 1,326 | 7.9 |  | 298 | 7.35 |
|  | Stage 2 | 3,670 | 21.85 |  | 930 | 22.94 |
|  | Stage 3 | 11,062 | 65.87 |  | 2,667 | 65.79 |
|  | Stage 4 | 735 | 4.38 |  | 159 | 3.92 |
| Stage at ART Start | |  |  |  |  |  |
|  | Stage 1 | 3,076 | 18.32 |  | 813 | 20.05 |
|  | Stage 2 | 2,423 | 14.43 |  | 612 | 15.1 |
|  | Stage 3 | 9,994 | 59.51 |  | 2,331 | 57.5 |
|  | Stage 4 | 1,300 | 7.74 |  | 298 | 7.35 |

Table S6. Logistic regression summary

| **Variable** | **Coefficient** | **p-value** |
| --- | --- | --- |
| Constant | -0.6935 | 1.0000 |
| Gender_Male | -0.1012 | 0.0310 |
| Regimen schedule_3-Month | -0.5705 | 1.0000 |
| Regimen schedule_6-Month | 0.7759 | 1.0000 |
| Regimen schedule_Regular | -0.8990 | 1.0000 |
| Prior ART_Naive | -0.2283 | 0.0000 |
| Method into ART_New | 0.1733 | 0.0020 |
| TB Rx Started_Yes | 0.0238 | 0.7190 |
| TPT Outcome_No treatement | -0.2868 | 1.0000 |
| TPT Outcome_Other | -0.2804 | 1.0000 |
| TPT Outcome_Rx completed | -0.1264 | 1.0000 |
| Regimen At Baseline_1S3E | -0.4602 | nan |
| Regimen At Baseline_1S3N | -0.1265 | nan |
| Regimen At Baseline_1T3E | -0.2327 | nan |
| Regimen At Baseline_1T3N | -0.0805 | nan |
| Regimen At Baseline_1Z3N | -0.1684 | nan |
| Regimen At Baseline_Other | 0.3748 | nan |
| Last ART Prescription_1T3E | 0.4445 | 1.0000 |
| Last ART Prescription_1T3O | -0.0952 | 1.0000 |
| Last ART Prescription_1Z3N | -0.5299 | 1.0000 |
| Last ART Prescription_2T3L | 0.1720 | 1.0000 |
| Last ART Prescription_2Z3L | -0.5956 | 1.0000 |
| Last ART Prescription_Other | -0.0893 | 1.0000 |
| Facility_CMC Coléah | -0.3278 | nan |
| Facility_CMC Flamboyants | 0.1955 | nan |
| Facility_CMC Matam | -0.5175 | nan |
| Facility_CMC Minière | 0.0114 | nan |
| Facility_CS Dabompa | 0.1347 | nan |
| Facility_CS Gbessia port 1 | -0.5642 | nan |
| Facility_CS Tombolia | -0.1978 | nan |
| Facility_CS Wanidara | 0.5721 | nan |
| TB Status At Last Visit_No symptoms | 0.0718 | 1.0000 |
| TB Status At Last Visit_Not Screened | 0.0708 | 1.0000 |
| TB Status At Last Visit_Other | -0.4090 | 1.0000 |
| TB Status At Last Visit_Screening Unknown | -0.4272 | 1.0000 |
| CPT at ART Start_Yes | 0.1086 | 0.0130 |
| Second Line Rx_Yes | -1.3247 | 0.0000 |
| Baseline CD4 | -0.0006 | 0.1140 |
| Last Pre-ART CD4 | 0.0008 | 0.0420 |
| Last ART CD4 | 0.0019 | 0.0000 |
| Last Pre-ART Stage | -0.0300 | 0.4460 |
| Stage at ART Start | -0.0824 | 0.0110 |
| Age At ART Start | -0.0051 | 0.4870 |
| Current Age | 0.0216 | 0.0040 |
| Duration on ART (months) | 0.0052 | 0.0000 |


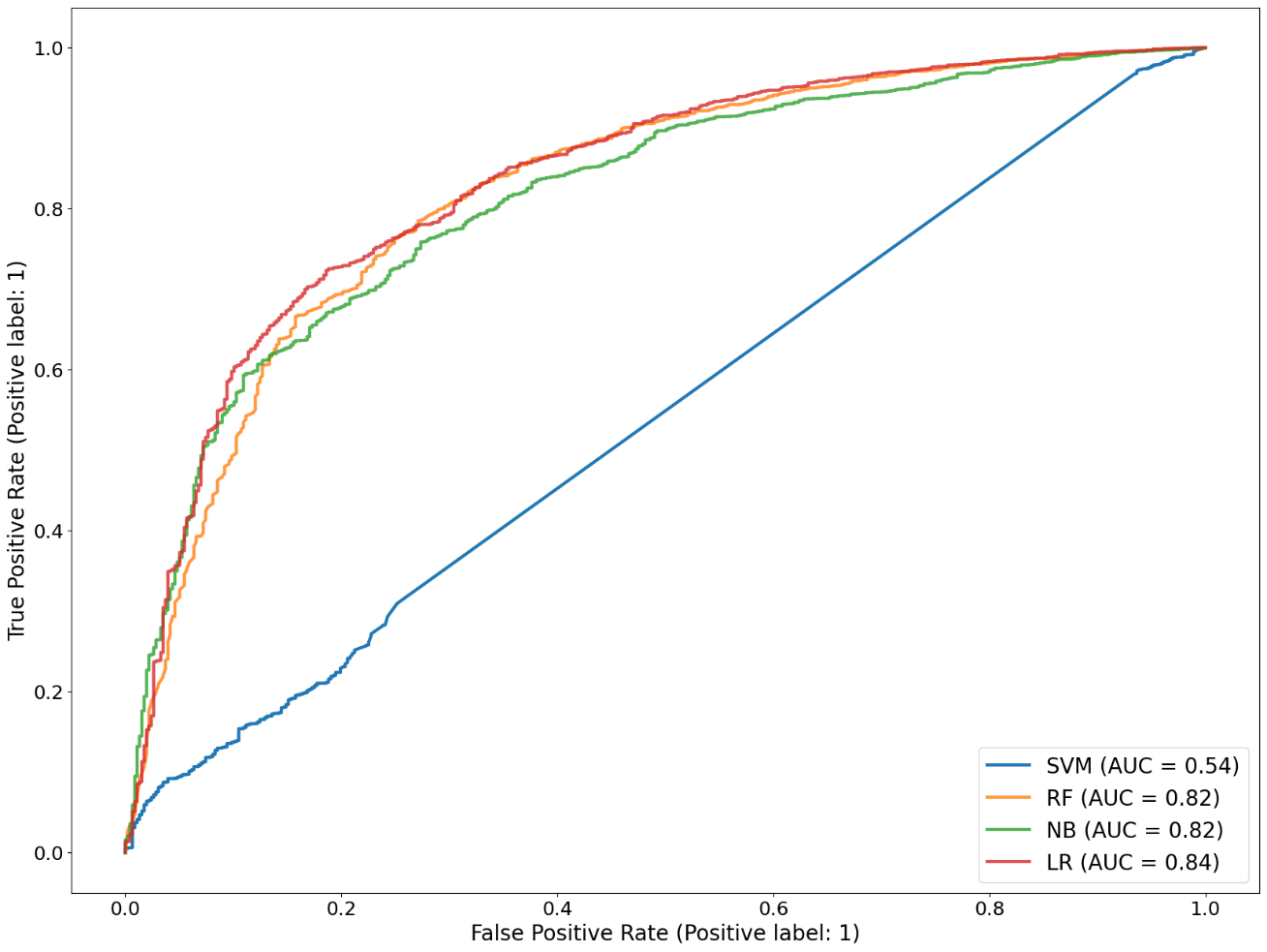


Figure S1. Areas under the curves of the four individual algorithms


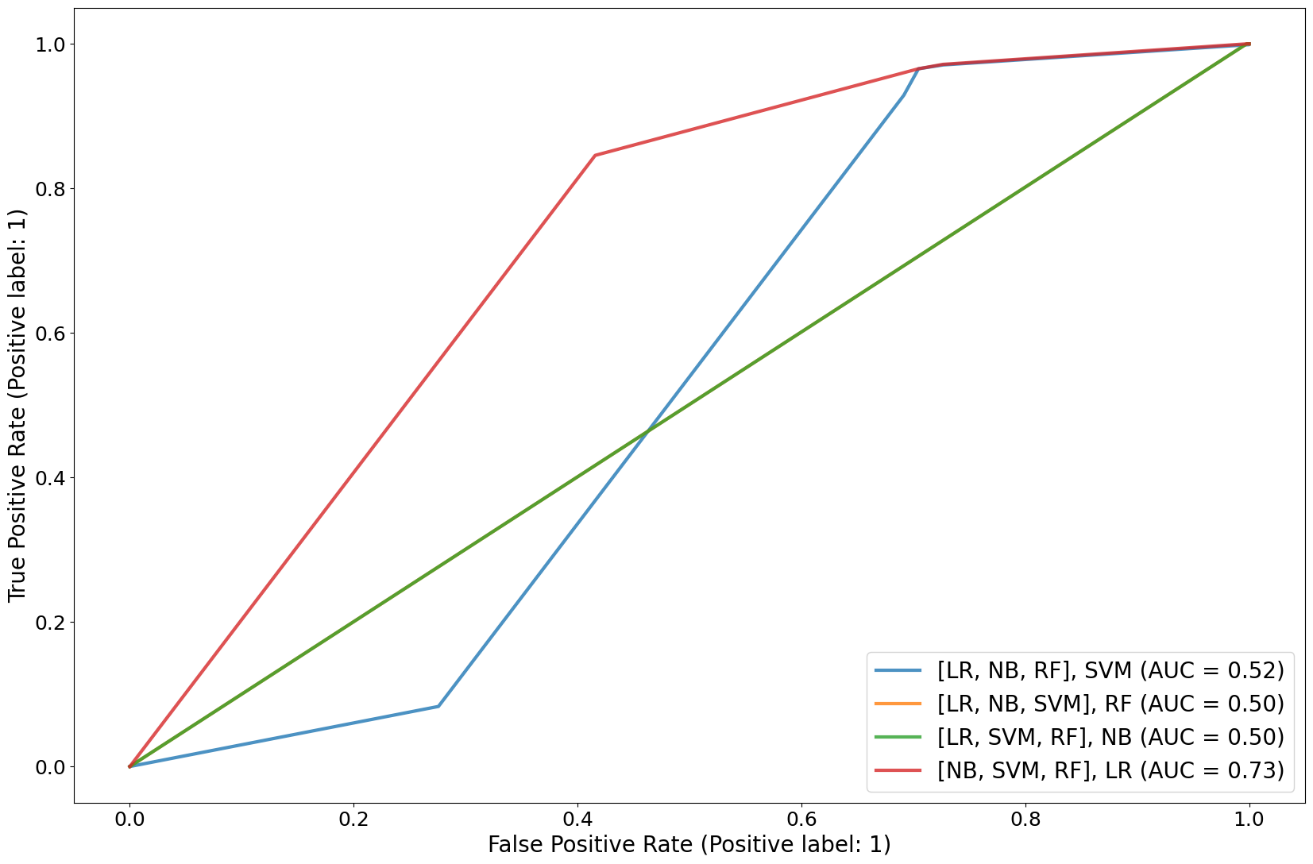


Figure S2. Areas under the curves of the four stacked algorithms
